# The Safety and Efficacy of Mechanical Thrombectomy with Acute Carotid Artery Stenting in an Extended Time Window: A Single-Center Study †

**DOI:** 10.3390/medsci14010047

**Published:** 2026-01-17

**Authors:** Bartosz Jabłoński, Adam Wyszomirski, Aleksandra Pracoń, Marcin Stańczak, Dariusz Gąsecki, Tomasz Gorycki, Waldemar Dorniak, Bartosz Regent, Michał Magnus, Bartosz Baścik, Edyta Szurowska, Bartosz Karaszewski

**Affiliations:** 1 Department of Adult Neurology, Faculty of Medicine, Medical University of Gdańsk, 80-210 Gdańsk, Poland; 2 Department of Adult Neurology, University Clinical Centre, 80-952 Gdańsk, Poland; 3 2nd Department of Radiology, Faculty of Health Sciences with the Institute of Maritime and Tropical Medicine, Medical University of Gdańsk, 80-210 Gdańsk, Poland; 4 Department of Radiology, University Clinical Centre, 80-952 Gdańsk, Poland; 5 Translational Brain Diseases Centre, The Fahrenheit Union of Universities, 80-210 Gdańsk, Poland

**Keywords:** tandem lesion, mechanical thrombectomy, emergent carotid artery stenting, antithrombotic treatment

## Abstract

**Background/Objectives**: Acute ischemic stroke (AIS) associated with cervical carotid artery pathology remains a therapeutic challenge due to uncertainty regarding emergent carotid artery stenting (eCAS) and the need for intensified antithrombotic therapy, which may increase the risk of hemorrhagic transformation (HT). This retrospective cohort study evaluated the functional and safety outcomes of eCAS within an extended treatment time window. **Methods**: We analyzed 139 consecutive patients with anterior circulation AIS and large vessel occlusion treated with mechanical thrombectomy between 2019 and 2024. Patients were eligible for MT within 24 h based on clinical–core mismatch (DAWN) or perfusion–core mismatch (DEFUSE 3) criteria. Outcomes were compared between patients treated with eCAS and those undergoing MT without stenting. **Results**: Twenty-five patients underwent eCAS, predominantly for tandem lesions (80%). Median age was 66 years, median baseline NIHSS was 14, and median infarct core volume on DWI/CTP was 15 mL. Baseline characteristics were comparable between groups, except for the site of occlusion (*p* < 0.001). A good functional outcome (modified Rankin Scale, mRS 0–2 at 90 days) was observed in 60% of patients in the eCAS group versus 43% in the non-stenting group, without statistical significance (*p* = 0.067). Rates of parenchymal hematoma (12% vs. 18.4%) and symptomatic intracerebral hemorrhage (8% vs. 3.5%) were similar between groups. **Conclusions**: In this single-center cohort, eCAS performed in an extended time window did not demonstrate a clear signal of increased hemorrhagic risk. However, residual confounding and imbalance between treatment groups persisted despite the application of inverse probability weighting (IPW), and the findings should be interpreted cautiously.

## 1. Introduction

Patients with acute ischemic stroke (AIS) and dual pathology represent approximately 15% of those undergoing mechanical thrombectomy (MT) [1]. The definition of dual pathology or “tandem lesion/occlusion” (TL) typically involves a proximal intracranial occlusion (distal ICA, M1-M2 MCA) along with an extracranial ICA lesion (high-grade stenosis/occlusion). The optimal management for this group is uncertain and depends on factors such as the etiology (atherosclerosis or dissection), center experience, and the interventionalist’s decision [2]. The main concern is the risk of hemorrhagic transformation (HT) associated with additional antithrombotic medication, especially dual antiplatelet therapy (DAPT) and glycoprotein IIb/IIIa inhibitors during emergent carotid artery stenting (eCAS). Careful patient selection for high-grade ICA stenosis or occlusion is crucial to determine the need for immediate eCAS versus delayed intervention.

Advancements in MT and expanded treatment criteria have allowed for more patients to be treated within an extended time window, as demonstrated in studies like DAWN [3], DEFUSE-3 [4], MR CLEAN LATE [5], and patients with large core AIS [6].

However, there is a lack of information on specific subgroups of patients with TL AIS, particularly those beyond the traditional 6 h time window, as these patients were not well-represented in the key MT trials. In the HERMES cohort, which includes patients from pivotal MT trials [7], TL AIS constituted only about 10% of all patients, making it challenging to provide clear recommendations based on that data. Additional information was obtained from the TITAN [8], ETIS [9], and STRATIS registries [10]. A combined analysis showed that eCAS is associated with higher odds of successful reperfusion and favorable outcomes compared to no extracranial carotid artery stenting, but with a higher risk of hemorrhagic complications. Recent data from the IRIS collaboration [11] includes patients with large vessel occlusion (LVO) who were primarily hospitalized in comprehensive stroke centers. In a secondary analysis focusing on patients with TL AIS (113 patients), there were no significant differences in outcomes between those who received eCAS during the MT procedure and those who did not, regardless of whether they received intravenous thrombolysis. However, it is worth noting that the definition of tandem lesions varied among the studies included in the analysis.

The latest study, CERES-TANDEM (NCT06965036), is the largest real-world analysis of eCAS in TL AIS patients during MT procedures. It involved 4053 patients from 49 stroke centers and showed improved outcomes with excellent and good functional results without additional harm, including sICH.

We are currently awaiting the results of several ongoing randomized clinical trials: TITAN (NCT03978988), EASI TOC (NCT04261478), START (NCT05902000), PICASSO (NCT05611242), and CASES (NCT06511089).

One of the most concerning complications of MT, especially when combined with eCAS and the need for additional antithrombotic therapy, is hemorrhagic transformation (HT). HT affects approximately 40% of patients undergoing MT [12], with symptomatic intracerebral hemorrhage (sICH) occurring in 5.4% of cases [13]. Risk factors for HT include a higher baseline NIHSS score, elevated initial glucose levels, older age, more severe early ischemic changes on neuroimaging (or ischemic core volume), poor collateral circulation status, history of hypertension, longer time from symptom onset to treatment, and more passes with the thrombectomy device [14,15]. Another major complication of eCAS is early reocclusion, which is estimated to occur in about 10–20% of cases and is heavily influenced by the antiplatelet strategy used [16].

In our AIS patient cohort with a high prevalence of dual pathology, we conducted a single-center study to assess the efficacy and safety of eCAS within an extended time window following the DAWN and DEFUSE-3 protocols. This study is particularly significant due to the non-standard time window, and there is a lack of data on the safety of this therapy in such cases.

## 2. Materials and Methods

### 2.1. Study Design

Preliminary data from this cohort were previously presented in abstract form at the World Stroke Congress [17].

This retrospective observational cohort study was conducted at a single-center to evaluate the effectiveness of eCAS in patients with AIS undergoing MT within an extended time window according to the DAWN and DEFUSE-3 criteria. The study included consecutive AIS patients admitted to the Stroke Unit of the Department of Adult Neurology at the University Clinical Center in Gdansk between 2019 and 2024, identified through the hospital’s information system. Patients were categorized into two groups based on whether they underwent eCAS during MT: the eCAS group and the non-stenting group. The primary objective was to compare functional and safety outcomes between these groups, focusing on the risk of hemorrhagic transformation (HT) after eCAS and the additional use of antithrombotic medications during the procedure.

Mechanical thrombectomy and emergent carotid artery stenting procedures were performed by a limited number of experienced neurointerventionalists (>50 MT procedures per year), at our high-volume comprehensive stroke center, where approximately 300 such procedures are performed annually.

To address potential biases from non-random treatment allocation, inverse probability weighting (IPW) using propensity scores was employed to adjust for differences in key baseline characteristics such as age, baseline NIHSS, core infarct volume on DWI/CTP, hypertension, site of occlusion, and time from onset to the groin. By utilizing IPW and adjusting for confounding variables, this study aimed to provide a more robust analysis of the impact of eCAS in AIS patients undergoing MT, considering the limitations of its observational design.

The study was approved by the local Ethics Committee of Medical University of Gdańsk, approval number KB/572/2025 (date of approval: 5 December 2025). Due to the retrospective nature of the study, the requirement for informed consent was waived.

This study was conducted and reported in accordance with the Strengthening the Reporting of Observational Studies in Epidemiology (STROBE) guidelines for cohort studies [18].

### 2.2. Participants

This prospective registry was conducted from 2019 to 2024 at the Stroke Unit of the Department of Adult Neurology at the University Clinical Center in Gdansk, a comprehensive stroke center serving the Pomeranian Region of Poland. The registry included 139 consecutive patients with AIS and LVO in the anterior circulation who were eligible for MT within 24 h based on clinical-core (DAWN) or perfusion-core mismatch (DEFUSE 3) criteria. All included patients were functionally independent prior to stroke onset, with a pre-stroke mRS scores of 0–2.

Exclusion criteria comprised posterior circulation stroke, absence of large vessel occlusion in the anterior circulation on baseline imaging, pre-stroke mRS score > 2, and mechanical thrombectomy performed outside the DAWN or DEFUSE-3 eligibility criteria.

Acute ischemic stroke was diagnosed based on standard clinical assessment supported by neuroimaging findings. As all patients in this cohort were evaluated in the extended time window, advanced neuroimaging techniques were mandatory in all cases in accordance with the DAWN and DEFUSE-3 protocols. The imaging workflow included non-contrast CT to exclude intracranial hemorrhage, CT angiography (CTA) to confirm large vessel occlusion, and CT perfusion (CTP) for assessment of ischemic core and penumbral tissue. In selected patients, magnetic resonance imaging (MRI) with diffusion-weighted imaging (DWI) and MR angiography (MRA) were also performed. Eligibility for mechanical thrombectomy was determined based on clinical–core or perfusion–core mismatch criteria as defined by the DAWN and DEFUSE-3 trials.

Wake-up strokes were included provided that patients fulfilled the DAWN or DEFUSE-3 eligibility criteria based on clinical–core or perfusion–core mismatch, irrespective of uncertainty regarding the exact time of symptom onset.

Due to the retrospective design of the study, the flowchart reflects the final analyzed cohort rather than all patients assessed for mechanical thrombectomy.

### 2.3. Data

The primary outcome was functional status at 90 days, assessed using the modified Rankin Scale (mRS). Good functional outcome was defined as mRS 0–2, and excellent outcome as mRS 0–1. The mRS evaluations were conducted by trained clinicians or research staff via standardized telephone interviews with patients or their caregivers.

Secondary outcomes included all-cause mortality at 90 days, the occurrence of parenchymal hematoma (PH) types 1 and 2, and symptomatic intracranial hemorrhage (sICH).

We identified a priori a set of potential confounders based on clinical relevance and literature, which could affect both treatment allocation (stenting vs. no-stenting) and outcomes. These confounders included: baseline National Institutes of Health Stroke Scale (NIHSS) score, age, core infarct volume measured by diffusion-weighted imaging (DWI) or cerebral blood flow (CBF) < 30% on CT perfusion (CTP), presence of hypertension, site of arterial occlusion, and time from onset to the groin.

We collected data on periprocedural antiplatelet therapy in relation to eCAS, including dual antiplatelet therapy, the use of glycoprotein IIb/IIIa inhibitors, and intravenous thrombolysis, as key pharmacological factors associated with the risk of hemorrhagic transformation.

In addition, we collected information on other baseline demographic and clinical variables: sex, prior intravenous thrombolysis, dyslipidemia, smoking status, type of admission (mothership vs. drip-and-ship), pre-treatment use of antithrombotic medications, diabetes mellitus, time from stroke symptom onset to treatment initiation and number of passes with the thrombectomy device. These variables were considered exploratory covariates and were assessed for group comparability.

The mismatch criteria were based on the DAWN and DEFUSE-3 protocols:

Perfusion-core mismatch:

- Infarct core volume < 70 mL
- critically hypoperfused volume/infarct core volume > 1.8
- mismatch volume > 15 mL
- Relative cerebral blood flow (rCBF) < 30% (CT perfusion), Tmax > 6 s

Clinical-core mismatch defined as one of the following on MRI-DWI or CTP-rCBF maps:

- <21 mL core infarct and NIHSS ≥ 10 (and age ≥ 80 years old)
- <31 mL core infarct and NIHSS ≥ 10 (and age < 80 years old)
- 31 mL to <51 mL core infarct and NIHSS ≥ 20 (and age < 80 years old)

A tandem lesion (TL) was defined as a proximal intracranial occlusion (distal ICA, M1-M2 MCA) along with an extracranial ICA lesion (high-grade stenosis > 70%/occlusion). Hemorrhagic complications were assessed using the ECASS III classification [19]. Control CT scans were conducted around 24 h post-treatment in all instances. Symptomatic intracerebral hemorrhage (sICH) was characterized by secondary hemorrhage of type PH2, either local or remote, within 22 to 36 h post-treatment (or sooner if clinical deterioration necessitates earlier imaging), along with a 4-point or greater rise in the NIHSS score.

### 2.4. Statistical Methods

No formal sample size calculation was performed for this study. The sample size was determined by the availability of patient data in the existing electronic health records from the Stroke Unit.

The modified Rankin Scale (mRS) scores were originally recorded as an ordinal numerical scale. For analysis and interpretability, the mRS was dichotomized into commonly used binary outcomes in stroke research: 0–1 versus 2–6 (excellent outcome), 0–2 versus 3–6 (good outcome), and 6 versus 0–5 (mortality).

Descriptive statistics for patient baseline characteristics were summarized. Continuous variables were reported as medians with interquartile ranges (IQR) due to expected non-normality and potential outliers, while categorical variables were described using absolute frequencies and percentages. Differences in continuous baseline variables between groups were assessed using the permuted Yuen-Welch t-test with Satterthwaite adjustment for degrees of freedom, incorporating 1000 replications and 10% trimming to account for unequal variances and the influence of outliers. Comparisons of categorical variables were performed using Fisher’s exact test.

Given the observational design and substantial imbalance between the eCAS (n = 25) and no-stenting (n = 114) groups, propensity score-based inverse probability weighting (IPW) was used to adjust for confounding and minimize selection bias. The propensity score model was a multivariable logistic regression estimating the probability of receiving emergent carotid artery stenting (eCAS) conditional on the following covariates: baseline NIHSS, age, core infarct volume measured by DWI/CBF < 30% CTP, hypertension, site of occlusion, time from onset to the groin, and number of passes with the thrombectomy device. Inverse probability weights were derived from these propensity scores. Covariate balance after weighting was evaluated using standardized mean differences (SMD), with values less than 0.10 indicating acceptable balance.

All outcomes were analyzed using weighted generalized linear models (GLMs) with a binomial distribution and identity link function, allowing estimation of risk differences (RDs) and 95% confidence intervals (CIs) comparing eCAS versus no-stenting groups. Additional covariate adjustment beyond weighting was not included in the final outcome models due to the small sample size and to avoid model overfitting and instability.

Statistical inference was based on Wald tests of model coefficients. A two-sided *p*-value < 0.05 was considered statistically significant. No correction for multiple comparisons was applied, given the exploratory nature of the study and the limited number of prespecified outcomes; results should therefore be interpreted cautiously.

The presence of missing values in the core DWI/CBF < 30% CTP variable was handled using the Stochastic Approximation Expectation-Maximization (SAEM) algorithm.

All statistical analyses were performed using R software, version 4.4.2 (R Core Team, 2019).

## 3. Results

### 3.1. Baseline Characteristics

**Figure 1:**
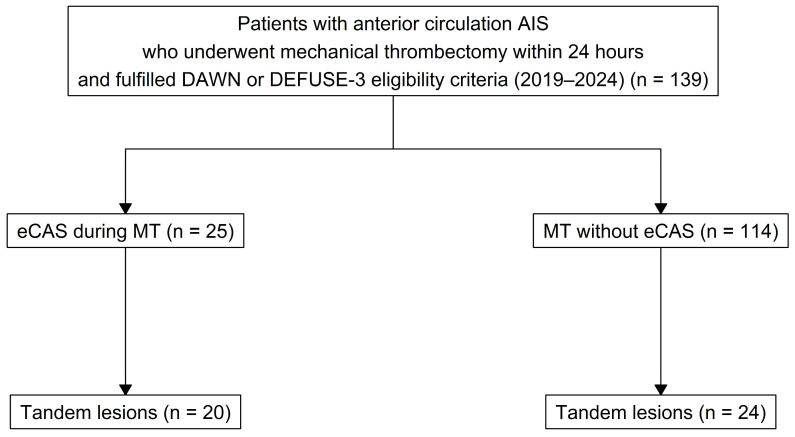
Flow chart.

The study included 25 patients who underwent emergent carotid artery stenting (eCAS) (24 of these also had angioplasty before stent placement) and 114 patients in the non-stenting cohort (Figure 1). The median age (Q1–Q3) was 69 (62–76) years, with 68 (48.9%) female patients in the overall cohort. Patients in the eCAS group had a significantly higher frequency of tandem occlusion (80% vs. 21.1%, *p* < 0.001) and atherosclerosis (88% vs. 17.5%, *p* < 0.001) compared to the non-stenting group. The median baseline NIHSS (Q1–Q3) was similar between the groups (14 (10–19) vs. 15.5 (12–19), *p* = 0.358), as was the time to treatment (14.1 (12.9–17.2) vs. 12.6 (10.3–15.5), *p* = 0.139) (Table 1).

In our cohort, 44 out of 139 patients (31.7%) had TL AIS, with 20 patients undergoing eCAS. In the non-stenting cohort, 20 patients (17.5%) underwent angioplasty during the mechanical thrombectomy (MT) procedure.

Among the eCAS group, 22 out of 25 patients (88.0%) received DAPT and/or glycoprotein IIb/IIIa inhibitor (eptifibatide), while 3 out of 25 patients (12.0%) received other medications (single antiplatelet therapy, unfractionated heparin).

### 3.2. Study Outcomes

The IPW model achieved good balance for age, hypertension, site of occlusion, and number of passes with thrombectomy device, with standardized mean differences (SMD) < 0.10. However, NIHSS score at admission (SMD = 0.261), core infarct volume measured by DWI/CBF < 30% CTP (SMD = 0.163), and time from onset to the groin (SMD = 0.287) remained above the prespecified threshold for acceptable balance.

**Figure 2:**
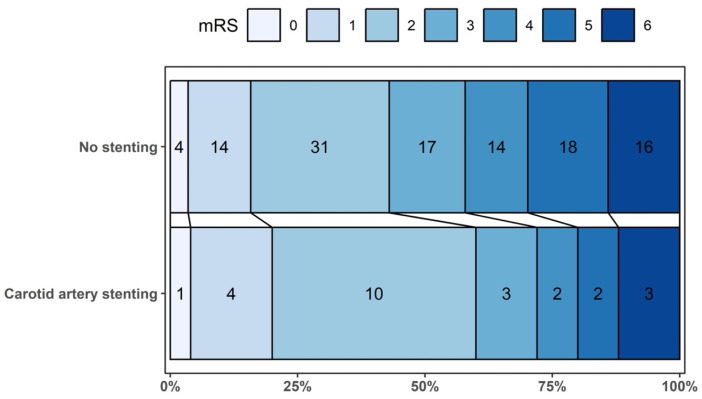
Distribution of participants according to their scores on the Modified Rankin Scale at 90 days.

Regarding the primary outcome, the proportion of patients achieving an excellent functional outcome (mRS 0–1 at 90 days) was 20% (5/25) in the eCAS cohort and 15.8% (18/114) in the non-stenting cohort (Figure 2). The risk difference (RD) for achieving an excellent outcome in the eCAS group compared to the non-stenting group was 0.05 (95% CI: −0.17; 0.26, *p* = 0.678, Table 2). For the good functional outcome (mRS 0–2) at 90 days, the proportions were 60% (15/25) in the eCAS group and 43% (49/114) in the non-stenting group. The RD for this outcome was 0.25 (95% CI: −0.02; 0.52, *p* = 0.067).

Mortality at 90 days was observed in 12% (3/25) of patients in the eCAS group and 14% (16/114) in the non-stenting group, with an associated risk difference of −0.03 (95% CI: −0.22; 0.16, *p* = 0.786). Parenchymal hematoma types 1 and 2 were observed in 12% (3/25) of patients in the eCAS group and 18.4% (21/114) in the non-stenting cohort. The corresponding RD for developing PH 1–2 in the eCAS cohort compared to the non-stenting group was −0.11 (95% CI: −0.31; 0.10, *p* = 0.308) (Table 2). Symptomatic intracranial hemorrhage (sICH) occurred in 8% (2/25) of patients in the CAS group and 3.5% (4/114) in the non-stenting cohort. The RD for sICH was 0.06 (95% CI: −0.06; 0.17, *p* = 0.317).

### 3.3. Subgroup Analysis

We compared the patient populations with tandem lesion acute ischemic stroke who underwent either eCAS or not (non-stenting group). 20% (4/20) of patients in the TL AIS eCAS group achieved an excellent functional outcome (mRS 0–1 at 90 days), compared to 16.7% (4/24) in the TL AIS non-stenting group. The risk difference for an excellent outcome in the eCAS group versus the non-stenting group was 0.03 (95% CI: −0.21; 0.27, *p* = 0.817) (Table 3). For a good functional outcome (mRS 0–2) at 90 days, 60% (12/20) of the eCAS group and 41.7% (10/24) of the non-stenting group achieved this result. The RD for this outcome was 0.18 (95% CI: −0.12; 0.49, *p* = 0.232). Mortality at 90 days was recorded in 10% (2/20) of the eCAS group and 8.3% (2/24) of the non-stenting group, with a risk difference of 0.02 (95% CI: −0.16; 0.20, *p* = 0.853). Parenchymal hematoma types 1 and 2 were found in 15% (3/20) of the eCAS group and 25% (6/24) of the non-stenting group. The RD for developing PH 1–2 in the eCAS group compared to the non-stenting group was −0.10 (95% CI: −0.34; 0.14, *p* = 0.416) (Table 3). Symptomatic intracranial hemorrhage (sICH) was observed in 10% (2/20) of the eCAS group and 4.2% (1/24) of the non-stenting group, with an RD of 0.06 (95% CI: −0.10; 0.22, *p* = 0.480).

## 4. Discussion

The management of tandem lesions in acute ischemic stroke poses a clinical challenge due to the lack of standardized recommendations and limited randomized studies in this area. Tandem lesions are associated with high mortality rates of up to 45% [20]. Clinical dilemmas include the choice of procedure technique, such as the sequence of thrombectomy and stenting, and the use of antithrombotic medications, which may increase the risk of secondary hemorrhage. Key trials such as the HERMES meta-analysis had a small number of tandem lesion patients and did not show significant outcome differences. The DAWN study did not allow stenting of extracranial lesions in tandem cases, while the DEFUSE-3 study had few patients who underwent extracranial carotid artery stenting. Current guidelines, AHA/ASA 2019 [21] or European Society for Minimally Invasive Neurological Therapy (ESMINT) Guidelines on Mechanical Thrombectomy in Acute Ischemic Stroke 2019 [2], do not provide definitive answers due to limited data.

A recent study by Anadani et al. [22] analyzed data from TITAN and ETIS registries showing that eCAS in tandem lesions resulted in higher rates of excellent and good functional outcomes, but with higher odds of any hemorrhage, excluding sICH or PH2. A meta-analysis by Diana et al. [23] also found that eCAS improved functional outcomes but increased the risk of sICH. Also, the CERES-TANDEM study demonstrated positive outcomes with eCAS in tandem lesions.

In our single-center study, the proportion of patients achieving excellent and good functional outcomes was similar between the eCAS and non-stenting groups. Safety parameters, such as 90-day mortality and the risk of PH1, PH2, and sICH, were also comparable. The overall rate of intravenous thrombolysis in our cohort was low. This is primarily attributable to the study’s focus on patients treated according to extended-window thrombectomy criteria, with a substantial proportion presenting beyond the approved time limits for IVT. The IVT rates observed in our cohort are comparable to those reported in the DAWN and DEFUSE-3 trials, where intravenous thrombolysis was administered in 5% and 11% of patients undergoing mechanical thrombectomy, respectively. The relatively low exposure to IVT in our study may have influenced the risk of hemorrhagic complications and should be considered when interpreting the safety outcomes. However, we acknowledge major limitations, which are discussed later in this text. This study is unique as it focuses on consecutive patients who underwent mechanical thrombectomy (MT) in the extended time window based solely on the DAWN and DEFUSE-3 qualifying criteria. A significant number of patients in the study had internal carotid artery (ICA) pathology. Of the total patients, 25 underwent eCAS, and in the non-stenting cohort, 20 patients (17.54%) received angioplasty during the MT procedure. In addition to atherosclerosis and rare cases of carotid dissection, occlusions in the non-stenting group were predominantly cardioembolic or embolic strokes of undetermined source. Etiology appeared to play an important role in treatment allocation, as emergent carotid artery stenting was performed primarily in patients with significant extracranial carotid atherosclerotic disease. This etiological imbalance between groups contributes to clinical heterogeneity and further limits causal inference regarding comparative outcomes.

One of the key uncertainties in the management of TL AIS is the use of antithrombotic medication, especially in cases with a longer time from symptom onset to treatment. Despite the frequent use of dual antiplatelet therapy and/or glycoprotein IIb/IIIa inhibitors in the eCAS group (88%), we did not observe a significantly higher incidence of sICH and PH1–2 HT compared with the non-stenting group.

### Limitations

This study has several limitations that should be considered when interpreting the findings.

First, the observational and non-randomized design of the study inherently introduces potential residual confounding, despite the application of inverse probability weighting (IPW) based on key baseline covariates. Although the propensity score model included important clinical and imaging factors, such as NIHSS baseline, age, core infarct volume, hypertension, site of occlusion, time from onset to the groin, and number of passes with the thrombectomy device, unmeasured or unknown confounders may still have influenced treatment allocation and outcomes.

Second, the observed numerical differences in hemorrhagic patterns between groups should be interpreted cautiously due to small event numbers. Parenchymal hematoma and symptomatic intracerebral hemorrhage represent distinct pathophysiological entities and may be influenced by different factors, including infarct evolution, reperfusion dynamics, and antithrombotic exposure.

Third, the limited sample size in the stenting group restricted the ability to perform multivariable adjustment within the outcome regression models beyond the use of IPW, to avoid model overfitting and instability.

Fourth, a formal sample size calculation was not conducted due to limited availability of patients, and no adjustments for multiple comparisons were made. These factors should be considered when interpreting the study’s findings.

Fifth, wake-up stroke status may have contributed to outcome heterogeneity but could not be analyzed as a separate subgroup owing to the limited sample size.

The lack of collateral assessment, which is an important factor in predicting hemorrhagic transformation after mechanical thrombectomy. Additionally, in our cohort, due to longer time to treatment (extended protocols mechanical thrombectomies), the percentage of patients receiving IVT was low (12% in eCAS group and 7% in non-stenting group), which may contribute to the overall risk of hemorrhagic complications. Treatment allocation was not randomized and depended on vascular anatomy, etiology, and operator judgment, which inevitably introduces selection bias despite statistical adjustment. The relatively long onset-to-treatment times observed in both groups are a direct consequence of the study design, which focused exclusively on patients treated in the extended time window according to DAWN and DEFUSE-3 criteria. Moreover, a substantial proportion of patients were managed within a drip-and-ship model, further contributing to treatment delays. These characteristics are inherent to extended-window cohorts and should be considered when interpreting both efficacy and safety outcomes.

## 5. Conclusions

In this single-center observational cohort, emergent carotid artery stenting performed within an extended time window did not demonstrate a clear signal of increased hemorrhagic risk, including hemorrhagic transformation or symptomatic intracerebral hemorrhage. However, given the retrospective design, small sample size, residual confounding despite the application of IPW, and heterogeneity between treatment groups, these findings should be interpreted with caution. Larger prospective and randomized studies are required to confirm the safety profile of eCAS and to better define its role in the management of patients with acute ischemic stroke and dual pathology treated beyond the conventional time window.

## Figures and Tables

**Table 1:** Baseline characteristics.

		Carotid Artery Stenting		
Variable ^1^	Overall N = 139	Yes N = 25	No N = 114	*p*-Value ^2^
**Age [years]**				0.117
Median (Q1–Q3)	69.0 (62.0–76.0)	66.0 (58.0–71.0)	70.0 (63.0–77.0)	
**Female, n/N (%)**	68/139 (48.9%)	10/25 (40.0%)	58/114 (50.9%)	0.381
**NIHSS baseline**				0.358
Median (Q1–Q3)	15.0 (11.0–19.0)	14.0 (10.0–19.0)	15.5 (12.0–19.0)	
**IVT, n/N (%)**	11/139 (7.9%)	3/25 (12.0%)	8/114 (7.0%)	0.416
**Site of occlusion, n/N (%)**				<0.001
Tandem occlusion	44/139 (31.7%)	20/25 (80.0%)	24/114 (21.1%)	
ICA extracranial	13/139 (9.4%)	5/25 (20.0%)	8/114 (7.0%)	
Other	82/139 (59.0%)	0/25 (0.0%)	82/114 (71.9%)	
**Time to treatment [hours]**				0.139
Median (Q1–Q3)	13.0 (10.5–15.9)	14.1 (12.9–17.2)	12.6 (10.3–15.5)	
**Core DWI/CBF < 30% CTP ^3^**				0.654
Median (Q1–Q3); N missing	19.0 (10.0–30.0); 11	18.0 (7.0–29.0); 2	20.0 (10.0–30.0); 9	
**Atherosclerosis, n/N (%)**				<0.001
Atherosclerosis	42/139 (30.2%)	22/25 (88.0%)	20/114 (17.5%)	
Dissection	5/139 (3.6%)	3/25 (12.0%)	2/114 (1.8%)	
No	92/139 (66.2%)	0/25 (0.0%)	92/114 (80.7%)	
**Antithrombotic medication, n/N (%)**				0.174
Antiplatelet	25/139 (18.0%)	3/25 (12.0%)	22/114 (19.3%)	
Oral anticoagulant	19/139 (13.7%)	1/25 (4.0%)	18/114 (15.8%)	
No	95/139 (68.3%)	21/25 (84.0%)	74/114 (64.9%)	
**Antithrombotic treatment during procedure, n/N (%)**				<0.001
No	103/139 (74.1%)	0/25 (0.0%)	103/114 (90.4%)	
DAPT and/or glycoprotein IIb/IIIa inhibitors	24/139 (17.3%)	22/25 (88.0%)	2/114 (1.8%)	
Others	12/139 (8.6%)	3/25 (12.0%)	9/114 (7.9%)	
**Hypertension, n/N (%)**	95/139 (68.3%)	14/25 (56.0%)	81/114 (71.1%)	0.159
**Diabetes mellitus, n/N (%)**	38/139 (27.3%)	8/25 (32.0%)	30/114 (26.3%)	0.622
**Dyslipidemia, n/N (%)**	54/139 (38.8%)	8/25 (32.0%)	46/114 (40.4%)	0.503
**Smoking, n/N (%)**	56/139 (40.3%)	10/25 (40.0%)	46/114 (40.4%)	>0.999
**Mothership, n/N (%)**				0.171
Yes	52/139 (37.4%)	6/25 (24.0%)	46/114 (40.4%)	
No, drip and ship	87/139 (62.6%)	19/25 (76.0%)	68/114 (59.6%)	
**Number of passes with thrombectomy device, n/N (%)**				0.543
1	74/139 (53.2%)	14/25 (56.0%)	60/114 (52.6%)	
2	40/139 (28.8%)	8/25 (32.0%)	32/114 (28.1%)	
3	12/139 (8.6%)	2/25 (8.0%)	10/114 (8.8%)	
4	9/139 (6.5%)	0/25 (0.0%)	9/114 (7.9%)	
5	3/139 (2.2%)	1/25 (4.0%)	2/114 (1.8%)	
6	1/139 (0.7%)	0/25 (0.0%)	1/114 (0.9%)	

^1^ Abbreviations: NIHSS, National Institutes of Health Stroke Scale; IVT, Intravenous Thrombolysis; DWI, Diffusion-Weighted Imaging; CBF, Cerebral Blood Flow; CTP, Computed Tomography Perfusion; DAPT, Dual Antiplatelet Therapy; Q1: the first quartile; Q3, the third quartile. ^2^ Permutation-based Yuen-Welch *t*-test for numerical variable and Fisher’s exact test for categorical variable. ^3^ Missing values due to being not assessed.

**Table 2:** Study outcomes.

	Carotid Artery Stenting		IPW-Weighted Binomial Regression with Identity Link	
Outcome	Yes N = 25	No N = 114	Risk Difference (95% CI)	*p*-Value
**mRS 0** **–** **1 at 90 days, n/N (%)**	5/25 (20.0%)	18/114 (15.8%)	0.05 (−0.17; 0.26)	0.678
**mRS 0** **–** **2 at 90 days, n/N (%)**	15/25 (60.0%)	49/114 (43.0%)	0.25 (−0.02; 0.52)	0.067
**Parenchymal hematoma (PH 1** **–** **2) vs. other hemorrhagic transformation types or none, n/N (%)**	3/25 (12.0%)	21/114 (18.4%)	−0.11 (−0.31; 0.10)	0.308
**Symptomatic intracranial hemorrhage, n/N (%)**	2/25 (8.0%)	4/114 (3.5%)	0.06 (−0.06; 0.17)	0.317
**Mortality at 90 days, n/N (%)**	3/25 (12.0%)	16/114 (14.0%);	−0.03 (−0.22; 0.16)	0.786

Abbreviations: mRS, Modified Rankin Scale; PH, Parenchymal hematoma; CI: Confidence interval; IPW: Inverse probability weighting.

**Table 3:** Study outcomes in the tandem occlusion subgroup.

	Carotid Artery Stenting		IPW-Weighted Binomial Regression with Identity Link	
Outcome	Yes N = 25	No N = 24	Risk Difference (95% CI)	*p*-Value
**mRS 0** **–** **1 at 90 days, n/N (%)**	5/25 (20.0%)	4/24 (16.7%)	0.03 (−0.21; 0.27)	0.817
**mRS 0** **–** **2 at 90 days, n/N (%)**	15/25 (60.0%)	10/24 (41.7%)	0.18 (−0.12; 0.49)	0.232
**Parenchymal hematoma (PH 1** **–** **2) vs. other hemorrhagic transformation types or none, n/N (%)**	3/25 (12.0%)	6/24 (25.0%)	−0.10 (−0.34; 0.14)	0.416
**Symptomatic intracranial hemorrhage, n/N (%)**	2/25 (8.0%)	1/24 (4.2%)	0.06 (−0.10; 0.22)	0.480
**Mortality at 90 days, n/N (%)**	3/25 (12.0%)	2/24 (8.3%)	0.02 (−0.16; 0.20)	0.853

Abbreviations: mRS, Modified Rankin Scale; PH, Parenchymal hematoma; CI: Confidence interval; IPW: Inverse probability weighting.

## Data Availability

The data supporting the findings of this study were obtained from the electronic hospital health record system of University Clinical Center, Gdańsk, Poland, and are not publicly accessible. A de-identified dataset underlying the results may be available from the corresponding author upon reasonable request.

## Abbreviations

The following abbreviations are used in this manuscript:

AIS	Acute ischemic stroke
eCAS	Emergent carotid artery stenting
DAPT	Dual antiplatelet therapy
HT	Hemorrhagic transformation
LVO	Large vessel occlusion
MT	Mechanical thrombectomy
TL	Tandem lesions
NIHSS	National Institutes of Health Stroke Scale
DWI	Diffusion-weighted imaging
CTP	Computed tomography perfusion
mRS	Modified Rankin Scale
sICH	Symptomatic intracerebral hemorrhage
CBF	Cerebral blood flow
rCBF	Relative cerebral blood flow
IQR	Interquartile range
IPW	Inverse probability weighting
SMD	Standardized mean difference
GLM	Generalized linear model
RD	Risk difference
CI	Confidence interval
SAEM	Stochastic Approximation Expectation-Maximization
Q1	The first quartile
Q3	The third quartile

## References

[1] Jadhav A.P., Zaidat O.O., Liebeskind D.S., Yavagal D.R., Haussen D.C., Hellinger F.R., Jahan R., Jumaa M.A., Szeder V., Nogueira R.G. (2019). Emergent Management of Tandem Lesions in Acute Ischemic Stroke: Analysis of the STRATIS Registry. Stroke.

[2] Turc G., Tsivgoulis G., Audebert H.J., Boogaarts H., Bhogal P., De Marchis G.M., Fonseca A.C., Khatri P., Mazighi M., Pérez de la Ossa N. (2022). European Stroke Organisation—European Society for Minimally Invasive Neurological Therapy Expedited Recommendation on Indication for Intravenous Thrombolysis before Mechanical Thrombectomy in Patients with Acute Ischaemic Stroke and Anterior Circulation Large Vessel Occlusion. Eur. Stroke J..

[3] Nogueira R.G., Jadhav A.P., Haussen D.C., Bonafe A., Budzik R.F., Bhuva P., Yavagal D.R., Ribo M., Cognard C., Hanel R.A. (2018). Thrombectomy 6 to 24 Hours after Stroke with a Mismatch between Deficit and Infarct. N. Engl. J. Med..

[4] Albers G.W., Marks M.P., Kemp S., Christensen S., Tsai J.P., Ortega-Gutierrez S., McTaggart R.A., Torbey M.T., Kim-Tenser M., Leslie-Mazwi T. (2018). Thrombectomy for Stroke at 6 to 16 Hours with Selection by Perfusion Imaging. N. Engl. J. Med..

[5] Olthuis S.G.H., Pirson F.A.V., Pinckaers F.M.E., Hinsenveld W.H., Nieboer D., Ceulemans A., Knapen R.R.M.M., Robbe M.M.Q., Berkhemer O.A., van Walderveen M.A.A. (2023). Endovascular Treatment versus No Endovascular Treatment after 6–24 h in Patients with Ischaemic Stroke and Collateral Flow on CT Angiography (MR CLEAN-LATE) in the Netherlands: A Multicentre, Open-Label, Blinded-Endpoint, Randomised, Controlled, Phase 3 Trial. Lancet.

[6] Liu C., Abdalkader M., Sang H., Sarraj A., Campbell B.C.V., Miao Z., Huo X., Yoo A.J., Zaidat O.O., Thomalla G. (2025). Endovascular Thrombectomy for Large Ischemic Core Stroke: A Systematic Review and Meta-Analysis of Randomized Controlled Trials. Neurology.

[7] Goyal M., Menon B.K., Van Zwam W.H., Dippel D.W.J., Mitchell P.J., Demchuk A.M., Dávalos A., Majoie C.B.L.M., Van Der Lugt A., De Miquel M.A. (2016). Endovascular Thrombectomy after Large-Vessel Ischaemic Stroke: A Meta-Analysis of Individual Patient Data from Five Randomised Trials. Lancet.

[8] Anadani M., Spiotta A., Alawieh A., Turjman F., Piotin M., Steglich-Arnholm H., Holtmannspötter M., Taschner C., Eiden S., Haussen D.C. (2019). Effect of Extracranial Lesion Severity on Outcome of Endovascular Thrombectomy in Patients with Anterior Circulation Tandem Occlusion: Analysis of the TITAN Registry. J. Neurointerv. Surg..

[9] Desilles J.P., Consoli A., Redjem H., Coskun O., Ciccio G., Smajda S., Labreuche J., Preda C., Ruiz Guerrero C., Mazighi M. (2017). Successful Reperfusion with Mechanical Thrombectomy Is Associated with Reduced Disability and Mortality in Patients with Pretreatment Diffusion-Weighted Imaging-Alberta Stroke Program Early Computed Tomography Score ≤6. Stroke.

[10] Mueller-Kronast N.H., Zaidat O.O., Froehler M.T., Jahan R., Aziz-Sultan M.A., Klucznik R.P., Saver J.L., Hellinger F.R., Yavagal D.R., Yao T.L. (2017). Systematic Evaluation of Patients Treated With Neurothrombectomy Devices for Acute Ischemic Stroke. Stroke.

[11] Cavalcante F., Treurniet K., Kaesmacher J., Kappelhof M., Rohner R., Yang P., Liu J., Suzuki K., Yan B., van Elk T. (2025). Intravenous Thrombolysis before Endovascular Treatment versus Endovascular Treatment Alone for Patients with Large Vessel Occlusion and Carotid Tandem Lesions: Individual Participant Data Meta-Analysis of Six Randomised Trials. Lancet Neurol..

[12] Nogueira R.G., Gupta R., Jovin T.G., Levy E.I., Liebeskind D.S., Zaidat O.O., Rai A., Hirsch J.A., Hsu D.P., Rymer M.M. (2015). Predictors and Clinical Relevance of Hemorrhagic Transformation after Endovascular Therapy for Anterior Circulation Large Vessel Occlusion Strokes: A Multicenter Retrospective Analysis of 1122 Patients. J. Neurointerv. Surg..

[13] Tian B., Tian X., Shi Z., Peng W., Zhang X., Yang P., Li Z., Zhang X., Lou M., Yin C. (2022). Clinical and Imaging Indicators of Hemorrhagic Transformation in Acute Ischemic Stroke after Endovascular Thrombectomy. Stroke.

[14] Sun J., Lam C., Christie L., Blair C., Li X., Werdiger F., Yang Q., Bivard A., Lin L., Parsons M. (2023). Risk Factors of Hemorrhagic Transformation in Acute Ischaemic Stroke: A Systematic Review and Meta-Analysis. Front. Neurol..

[15] Kaesmacher J., Kaesmacher M., Maegerlein C., Zimmer C., Gersing A.S., Wunderlich S., Friedrich B., Boeckh-Behrens T., Kleine J.F. (2017). Hemorrhagic Transformations after Thrombectomy: Risk Factors and Clinical Relevance. Cerebrovasc. Dis..

[16] Poppe A.Y., Jacquin G., Roy D., Stapf C., Derex L. (2020). Tandem Carotid Lesions in Acute Ischemic Stroke: Mechanisms, Therapeutic Challenges, and Future Directions. AJNR Am. J. Neuroradiol..

[17] Jabłoński B., Wyszomirski A., Pracoń A., Stańczak M., Gąsecki D., Karaszewski B. (2025). The safety and efficacy of mechanical thrombectomy with acute carotid artery stenting in an extended time window: A single- center study. Int. J. Stroke.

[18] Vandenbroucke J.P., von Elm E., Altman D.G., Gøtzsche P.C., Mulrow C.D., Pocock S.J., Poole C., Schlesselman J.J., Egger M. (2007). Strengthening the Reporting of Observational Studies in Epidemiology (STROBE): Explanation and Elaboration. PLoS Med..

[19] Hacke W., Kaste M., Bluhmki E., Brozman M., Dávalos A., Guidetti D., Larrue V., Lees K.R., Medeghri Z., Machnig T. (2008). Thrombolysis with Alteplase 3 to 4.5 Hours after Acute Ischemic Stroke. N. Engl. J. Med..

[20] Pires Coelho A., Lobo M., Gouveia R., Silveira D., Campos J., Augusto R., Coelho N., Canedo A. (2020). Overview of Evidence on Emergency Carotid Stenting in Patients with Acute Ischemic Stroke Due to Tandem Occlusions: A Systematic Review and Meta-Analysis. J. Cardiovasc. Surg. (Torino).

[21] Powers W.J., Rabinstein A.A., Ackerson T., Adeoye O.M., Bambakidis N.C., Becker K., Biller J., Brown M., Demaerschalk B.M., Hoh B. (2019). Guidelines for the Early Management of Patients with Acute Ischemic Stroke: 2019 Update to the 2018 Guidelines for the Early Management of Acute Ischemic Stroke a Guideline for Healthcare Professionals from the American Heart Association/American Stroke Association. Stroke.

[22] Anadani M., Marnat G., Consoli A., Papanagiotou P., Nogueira R.G., Siddiqui A., Ribo M., Spiotta A.M., Bourcier R., Kyheng M. (2021). Endovascular Therapy of Anterior Circulation Tandem Occlusions: Pooled Analysis From the TITAN and ETIS Registries. Stroke.

[23] Diana F., Romoli M., Toccaceli G., Rouchaud A., Mounayer C., Romano D.G., Di Salle F., Missori P., Zini A., Aguiar De Sousa D. (2023). Emergent Carotid Stenting versus No Stenting for Acute Ischemic Stroke Due to Tandem Occlusion: A Meta-Analysis. J. Neurointerv. Surg..

